# Natural Warriors against Stored-Grain Pests: The Joint Action of *Beauveria bassiana* and *Steinernema carpocapsae*

**DOI:** 10.3390/jof9080835

**Published:** 2023-08-08

**Authors:** Waqas Wakil, Nickolas G. Kavallieratos, Nikoleta Eleftheriadou, Taha Yaseen, Khawaja G. Rasool, Mureed Husain, Abdulrahman S. Aldawood

**Affiliations:** 1 Department of Entomology, University of Agriculture, Faisalabad 38040, Pakistan; mtyh786@gmail.com; 2 Senckenberg German Entomological Institute, D-15374 Müncheberg, Germany; 3 Laboratory of Agricultural Zoology and Entomology, Department of Crop Science, Agricultural University of Athens, 75 Iera Odos street, 11855 Athens, Greece; nikolelef@aua.gr; 4 Department of Plant Protection, College of Food and Agriculture Sciences, King Saud University, P.O. Box 2460, Riyadh 11451, Saudi Arabia; gkhawaja@ksu.edu.sa (K.G.R.); mbukhsh@ksu.edu.sa (M.H.); aldawood@ksu.edu.sa (A.S.A.)

**Keywords:** stored-product insects, nematodes, fungi, biocontrol agent, sustainability, IPM

## Abstract

*Tribolium castaneum*, *Trogoderma granarium*, *Oryzaephilus surinamensis*, *Sitophilus oryzae*, *Rhyzopertha dominica*, and *Cryptolestes ferrugineus* are all major pests of stored grains. In this study, the efficiency of single and joint applications of the entomopathogenic nematode (EPN) *Steinernema carpocapsae* at two different doses (50 and 100 IJs cm^−2^) and the entomopathogenic fungus (EPF) *Beauveria bassiana* for the management of the aforementioned pests was estimated. At single treatments, both doses of *S. carpocapsae* caused higher mortality rates to all six pest species compared to *B. bassiana*. The combined treatment of EPF and EPN resulted in higher mortality compared to single treatments. Mortality was strongly influenced by the exposure interval and the application dose of the EPN at both single and combined treatments. Maximum mortality was observed for the application of the combined treatment at the high dose of *S. carpocapsae* and *B. bassiana*. Among the different insect species tested, the maximum mortality rate was observed for *R. dominica* (96.62%), followed by *S. oryzae* (90.48%), *T. castaneum* (87.23%), *C. ferrugineus* (76.05%), *O. surinamensis* (70.74%), and *T. granarium* (57.71%). The outcomes of this study demonstrate the potential of utilizing specific combinations of EPF and EPN as effective natural enemies against stored-grain pests.

## 1. Introduction

A multitude of insect pests pose a threat to stored commodities, causing considerable grain losses during the storage period [1]. Apart from the direct damage to grains, pest infestation results in the presence of extraneous matter in stored products, such as insect pests and their fragments, and insect excreta, that compromises the product quality and renders it unsuitable for human consumption [2]. Numerous species of stored-product pests exhibit a cosmopolitan distribution and can infest a wide range of stored grains and food. *Tribolium castaneum* (Herbst) (Tenebrionidae: Coleoptera) is a secondary pest of various stored products around the world [3,4], infesting up to 233 different commodities [5]. *Trogoderma granarium* Everts (Dermestidae: Coleoptera) is a primary pest that has been listed as one of the 100 worst invasive species globally [6], and is classified in the A_1_ quarantine category of the EPPO [7]. *Oryzaephilus surinamensis* (L.) (Silvanidae: Coleoptera) is also a severe cosmopolitan secondary pest of a range of grain products, as well as dried fruits and nuts [5,8,9]. *Sitophilus oryzae* (L.) (Curculionidae: Coleoptera) is a widely distributed primary pest and can deteriorate both the quality and quantity of grains [5,9]. *Rhyzopertha dominica* (F.) (Bostrychidae: Coleoptera) is a primary insect pest of cereals, corn bread, rice, and wheat, among other commodities across the world, feeding directly on the germ and endosperm of grains [5,10], while adults can fly between agricultural and non-agricultural areas [11]. Finally, *Cryptolestes ferrugineus* (Stephens) (Laemophloeidae: Coleoptera) is a ubiquitous and highly destructive secondary pest of a wide range of stored grains and products, causing great economic losses [12,13,14].

Currently, the application of organophosphates, pyrethroids, and fumigants represents the dominant approach for managing arthropod infestations in stored products [15]. However, the indiscriminate and excessive use of these synthetic insecticides has generated the development of resistance in storage pests, undermining the effectiveness of pest management strategies [15,16,17,18]. The development of tolerance to synthetic pesticides coupled with their significant residues and adverse effects on the environment and human health [15,19,20,21] has led the research community to the exploration of alternative methods, such as biopesticides, as crucial components in integrated pest management (IPM) approaches [22]. Biopesticides are natural products implementing living organisms, e.g., plants, nematodes, minerals, bacteria, fungi, and viruses, and are used to control or reduce pest populations [22]. 

Among the biological control agents, entomopathogenic fungi (EPF) have demonstrated their potential against various stored-grain insect pests [23,24,25,26]. Over 750 species of fungi are known to be entomopathogens [27]. For several of them, their utilization has been suggested to control stored-grain insect pests due to their safety towards humans, mammals, and the environment [28,29]. In insects, fungal infection initiates with the attachment of fungal conidia to the insect, followed by their penetration through the cuticle by secreting cuticle-degrading enzymes [30,31]. Subsequently, hyphae colonize the insect’s body and release mycotoxins, leading to the demise of the target insect pest within a few days [31,32]. To date, several species of EPF have been proven highly effective against a spectrum of insect pests [24,33,34,35].

Another biological control agent, entomopathogenic nematodes (EPNs), mainly belonging to *Steinernema* spp. and *Heterorhabditis* spp., have been proven highly effective against a variety of insect pests [36,37]. The parasitic life cycle of nematodes begins with the infestation of the insect host by the non-feeding third-stage infective juveniles (IJs), which enter through natural body openings such as the spiracles, anus, mouth, or the cuticle [36,37,38]. The genera *Steinernema* and *Heterorhabditis* carry symbiotic bacteria, i.e., *Xenorhabdus* spp. and *Photorhabdus* spp., respectively [37,39,40]. Once inside the host, IJs penetrate the layer of intestinal or tracheal epithelial cells to access the hemocoel, where they release symbiotic bacteria. The released bacteria rapidly multiply, causing septicemia and toxemia, leading to the host’s demise within 24–48 h. Symbiotic bacteria also serve as a food source for the EPNs, aiding in their maturation into adults [36,37,41,42]. Within the host cadaver, two to three generations of nematodes are typically completed [37,41]. Upon exhaustion of food reserves, the nematode offspring is released, developing into IJs that are able to survive in the environment, seeking out new hosts [36,37,41]. Since the initial discovery of highly effective EPNs of *Steinernema* spp. and *Heterorhabditis* spp. in the early 1990s, there has been growing interest in the research community, leading to increased study and investigation of these organisms [43,44,45,46,47,48].

The efficiency of biopesticides can be enhanced by the combined application of different biocontrol agents, rather than a single agent [49]. In this context, several studies have explored the effectiveness of the combination of EPF and EPNs towards various field and forest pests [49,50,51,52,53,54,55,56]. Nevertheless, two historic studies examined the efficacy of *Steinernema carpocapsae* (Weiser) (Rhabditida: Steinernematidae) with *Beauveria bassiana* (Bals. -Criv.) Vuill. (Hypocreales: Cordycipitaceae) [57], and *S. carpocapsae* with *Cordyceps farinosa* (Holmsk.) Kepler, B. Shrestha and Spatafora (Hypocreales: Cordycipitaceae) [58] as a treatment against larvae of stored-product insects. However, there are no data on the evaluation of *B. bassiana* and *S. carpocapsae* alone or in conjunction against a wide spectrum of adult stored-product insects. Therefore, the current study deals with the previously unexplored combined efficacy of *B. bassiana* and *S. carpocapsae* against adults of *T. castaneum*, *T. granarium*, *O. surinamensis*, *S. oryzae*, *R. dominica*, and *C. ferrugineus* under different doses and exposure intervals. The single applications of *B. bassiana* and *S. carpocapsae* against the aforementioned pests were also tested.

## 2. Materials and Methods

### 2.1. Rearing of Insects

Populations of *T. castaneum*, *T. granarium*, *O. surinamensis*, *R. dominica*, *S. oryzae*, and *C. ferrugineus* were acquired from the Microbial Control Laboratory, Department of Entomology, University of Agriculture, Faisalabad, where they were cultured without any subjection to insecticides for more than 10 years. *Trogoderma granarium* <24 h old adults were used in all bioassays. For *R. dominica*, *C. ferrugineus*, *T. castaneum*, *S. oryzae*, and *O. surinamensis*, <2 weeks old adults were used. The populations of *R. dominica*, *T. granarium*, and *S. oryzae* were reared on wheat at 30 °C, 65% relative humidity (RH) in complete darkness [59,60]. The populations of *C. ferrugineus* were cultured on wheat flour. Populations of *T. castaneum* were reared on wheat flour with 5% brewer’s yeast at the same environmental conditions as above [60]. Finally, *O. surinamensis* populations were reared on partially broken wheat grains, oat flakes, and brewer’s yeast powder (ratio 5:5:1) at the aforementioned conditions [61].

### 2.2. Culturing EPF

*Beauveria bassiana* (isolate WG-13) was taken from the collection of the Microbial Control Laboratory, Department of Entomology, University of Agriculture, Faisalabad. For mass cultivation of the culture, *B. bassiana* was inoculated to the growth medium Sabouraud Dextrose Agar (SDA) (Sigma-Aldrich Chemie GmbH, Taufkirchen, Germany) in Petri dishes (10 cm diameter), sheathed using parafilm and introduced in an incubator at 25 °C with light 14 h: dark 10 h photoperiod for 10 days. After incubation, *B. bassiana* conidia were scraped from the media dishes using a sterile scalpel and introduced in 50 mL falcon tubes containing 30 mL of surfactant solution (0.05%) (Silwet^TM^ L-77). For complete homogenization, the solution was vortexed for 5 min, after the addition of eight glass beads to aid agitation [56,62]. The conidial concentration of 1 × 10^6^ conidia mL^−1^ was determined under a hemocytometer (Neubauer-improved, Marienfeld, Lauda-Königshofen, Germany) with the use of a microscope (BB.1152-PLi, Euromex Microscopen bv, Arnhem, The Netherlands). Germination of conidia was determined by inoculating a dish (6 cm diameter) containing a mixture of yeast (1%) and SDA with 0.1 mL of conidial solution (1 × 10^6^ conidia mL^−1^). The dish was secured with parafilm and introduced in an incubator at 25 °C for 16 h, under light 14 h: dark 10 h [56,62]. Subsequently, the germination was determined by counting a total of 200 conidia inside the dish under a microscope at 400× magnification, and >91% germination was ensured prior to each assay [63].

### 2.3. Culturing EPN

The *Steinernema carpocapsae* (ALL strain) culture, originating from the collection of USDA-ARS (Byron, GA, USA) was obtained from the Microbial Control Laboratory, Department of Entomology, University of Agriculture, Faisalabad. The culture was reared on the larvae (last instar) of *Galleria mellonella* (L.) (Lepidoptera: Pyralidae). The infective juveniles (IJs) were harvested using white traps placed in Petri dishes (10 cm) [64]. Nematodes were stored at 14 °C in distilled water inside tissue culture flasks (250 mL). EPNs stored for <2 weeks were utilized for the bioassays. Two different concentrations, 50 and 100 IJs cm^−2^ (=500 and 1000 IJs mL^−1^), were determined for the bioassays using a Malassez hemocytometer (Bioanalytic GmbH, Umkirch, Germany) [56].

### 2.4. Bioassay

Single and combined application of the biological control agents (*B. bassiana* and *S. carpocapsae*) were tested under laboratory conditions. The bioassay arena consisted of filter papers (Whatman^TM^, 10 cm diameter, 87 g m^−2^ basis weight, 180 µm thickness) placed inside Petri dishes (10 cm diameter). Polytetrafluoroethylene (Sigma-Aldrich Chemie GmbH in Taufkirchen, Germany) was applied to the edges of the dishes to prevent insects from escaping. For the single-treatment application of *S. carpocapsae*, 1 mL of each dose of *S. carpocapsae* (500 and 1000 IJs mL^−1^) was applied on the filter paper of separate dishes using a pipette. Subsequently, 20 adults of each insect species were released in separate dishes for each of the two *S. carpocapsae* concentrations. For the single-treatment application of *B. bassiana*, the filter paper was pipetted with a 1 mL conidial suspension of *B. bassiana* (1 × 10^6^ conidia mL^−1^) [65]. The filter paper inside the Petri dish was permitted to soak for 30 s. Next, 20 individuals of each insect species were released inside separate dishes, using new *B. bassiana* suspensions. For the combined treatment of *B. bassiana* and *S. carpocapsae*, *B. bassiana* was applied first by pipetting, as described above, followed by the application of *S. carpocapsae* according to the aforementioned protocol. Separate dishes were prepared for the joint treatment of *B. bassiana* and *S. carpocapsae* for each dose of *S. carpocapsae*. Following this, 20 adults of each pest species were introduced inside each dish, with the implementation of new *B. bassiana* suspensions. A single dish was treated with distilled water plus Silwet^TM^ L-77 (0.05%) to serve as a control. All dishes were kept at 30 °C and 65% RH in darkness until assessment. Mortality was recorded at 3, 7, and 14 days post-exposure for each of the single and combined treatments, where new series of Petri dishes were prepared per exposure. The experiment consisted of five treatments in total (single and combined), and each treatment was repeated thrice. The entire bioassay was repeated twice in total (2 replicates × 3 sub-replicates), with the implementation of fresh material (dishes, insects, *B. bassiana* and *S. carpocapsae* suspensions) for each replication. To verify that the cause of insect mortality was due to fungal infection for the single treatment with *B. bassiana*, after each mortality check, insect cadavers were sanitized by applying a solution of sodium hypochlorite (0.05%) for 3 min, and then washed thrice using distilled water. Later, dead insects were placed on SDA dishes at 25 °C and 75% RH for 7 days. The emergence of conidial spores from the insect cadavers was examined using a stereomicroscope [66]. For the single treatment of *S. carpocapsae,* dead individuals of each pest species were removed from the Petri dishes and dissected under a stereomicroscope to validate the existence of *S. carpocapsae* within their bodies [67]. Concerning the combined treatments, the presence of conidial spores was ascertained first, according to the aforementioned protocol, followed by the validation of the presence of *S. carpocapsae* as described above.

### 2.5. Statistical Analysis

Abbott’s formula was used for the correction of mortality among the treatment groups compared to the control group [68]. Prior to analysis, the values were transformed by the logarithm of (x + 1) to normalize the variance [69,70]. For the bioassays, values were analyzed by a two-way ANOVA for each pest tested. Exposure and treatment were the main effects and mortality was the response variable. The Tukey-Kramer (HSD) test was employed to distinguish means at 0.05 level of significance [71]. All analyses were conducted in Minitab software 2017 [72].

## 3. Results

### 3.1. Rhyzopertha dominica Mortality

All main effects and their associated interaction significantly affected the mortality of *R. dominica* (Table 1). Similar significant effects were observed for all the insect species tested. The joint application of both *B. bassiana* and *S. carpocapsae* at the high dose exhibited higher mortality at all exposure intervals compared to single treatments. Among all exposure intervals, the single application of *S. carpocapsae* caused greater mortality rates at both doses compared to single application of *B. bassiana*. At 3 days post-exposure, all treatments caused <35% mortality. The maximum observed mortality was 34.47% for the joint treatment of *B. bassiana* and *S. carpocapsae* at 100 IJs cm^−2^. At 7 days post-exposure, single treatments did not exceed 37% mortality, while combined treatments resulted in 58.42% and 71.22% for the low and high doses of *S. carpocapsae*, respectively. After 14 days, the combined treatments exhibited statistically higher efficacy against all single treatments, irrespective of the dose. Maximum mortality (96.62%) was observed for the combination of *B. bassiana* and *S. carpocapsae* at 100 IJs cm^−2^, followed by the same combination at the low dose (82.85%), application of *S. carpocapsae* alone at the high (51.22%) or low dose (43.59%), and *B. bassiana* alone (36.71%) (Table 2).

### 3.2. Sitophilus oryzae Mortality

The joint application of *B. bassiana* and *S. carpocapsae* at the high dose exhibited higher mortality at all exposure intervals compared to single ones. Irrespective of exposure interval, among the single treatments, the application of *S. carpocapsae* at its high dose caused higher mortality, followed by *S. carpocapsae* at the low dose, and *B. bassiana*. At 3 days post-exposure, no treatment was able to cause more than 30% mortality. The maximum observed mortality was 27.85% for the conjunction of *B. bassiana* and *S. carpocapsae* at its high dose. At 7 days post-exposure, single treatments did not exceed 35% mortality, with the maximum mortality observed for the high dose of *S. carpocapsae* (32.45%). Regarding the combined treatments, mortality was >50%. The maximum observed mortality reached 65.00% for the high dose of *S. carpocapsae*. After 14 days of exposure, all single treatments were unable to cause >47% mortality. Regardless of the dose, joint treatments demonstrated significantly higher effectiveness compared to single treatments. The maximum observed mortality was 90.48% for the joint application of *B. bassiana* and the high dose of *S. carpocapsae,* followed by the combination of *B. bassiana* and *S. carpocapsae* at the low dose (75.83%), single application of *S. carpocapsae* at a high (46.57%) or low dose (38.86), and single treatment of *B. bassiana* (32.71%) (Table 3).

### 3.3. Tribolium castaneum Mortality

The conjunction of *B. bassiana* and *S. carpocapsae* at the high dose resulted in higher mortality at all exposure intervals compared to the single treatments. Among single treatments, the application of *S. carpocapsae* at the high dose caused higher mortality, followed by *S. carpocapsae* at the low dose, and *B. bassiana*. After 3 days of exposure, the maximum observed mortality was 26.79% for the joint application of *B. bassiana* and *S. carpocapsae* at the high dose. At 7 days post-exposure, the joint treatment of *B. bassiana* and *S. carpocapsae* at 100 IJs cm^−2^ reached 60.17% mortality, which was the maximum observed mortality, significantly higher than all others. Fourteen days post-exposure, the joint treatments at both doses exhibited statistically higher mortalities compared to single ones. The joint treatment of *B. bassiana* and *S. carpocapsae* at the high dose exhibited the highest mortality (87.23%), followed by the combined treatment at the low dose (71.71%), *S. carpocapsae* alone at the high dose (43.55%), *S. carpocapsae* alone at the low dose (36.62%), and the single application of *B. bassiana* (27.28%) (Table 4).

### 3.4. Trogoderma granarium Mortality

The combined application of biocontrol agents produced higher mortality at all exposure intervals compared to single treatments. Among all exposure intervals, the single application of *S. carpocapsae* caused higher mortality compared to *B. bassiana* alone. After 3 days of exposure, all treatments caused <13% mortality. The maximum observed mortality was 12.63% for the conjunction of *B. bassiana* plus *S. carpocapsae* at the high dose, while the single application of *B. bassiana* did not affect the mortality of *T. granarium* (0.00%). At 7 days post-exposure, the combined treatment including the high dose of *S. carpocapsae* and *B. bassiana* caused the highest observed mortality (35.08%). At the final exposure interval (14 days), both combined treatments (high and low dose of *S. carpocapsae* and *B. bassiana*) resulted in statistically higher mortality compared to single treatments. The maximum observed mortality was 57.71% for the conjunction of *B. bassiana* and the high dose of *S. carpocapsae*, followed by the same combined treatment at the low dose of *S. carpocapsae* (41.36%), the single application of *S. carpocapsae* at the high dose (33.55%), and *S. carpocapsae* alone at the low dose (29.25%). The single application of *B. bassiana* remained the least effective (13.77%) (Table 5).

### 3.5. Cryptolestes ferrugineus Mortality 

The conjunction of *B. bassiana* and *S. carpocapsae* produced higher mortality at all exposure intervals compared to single treatments. In the single treatments, *S. carpocapsae* caused higher mortality than *B. bassiana* for all exposures. At 3 days of exposure, treatments caused <19% mortality. The maximum observed mortality was 18.50% for the joint application of *B. bassiana* and *S. carpocapsae* at the high dose. Seven days post-exposure, the conjunction of *B. bassiana* with the high dose of *S. carpocapsae* caused 51.71% mortality of *C. ferrugineus*. At the exposure of 14 days, regardless of the dose, joint treatments exhibited statistically higher mortalities compared to single treatments. The maximum observed mortality was 76.05% for the joint application of *B. bassiana* and *S. carpocapsae* at the high dose, followed by the same combined treatment at the low dose of *S. carpocapsae* (61.57%), single treatment with *S. carpocapsae* at the high (38.42%) or low dose (27.23%), and *B. bassiana* alone (19.56%) (Table 6).

### 3.6. Oryzaephilus surinamensis Mortality

The joint application of *B. bassiana* and *S. carpocapsae* exhibited higher mortality at all exposure intervals than single treatments. Concerning the single treatments, the application of *S. carpocapsae* at both doses caused higher mortality rates compared to *B. bassiana* for all exposures. At 3 days post-exposure, the maximum observed mortality was 17.7% for the joint application of *B. bassiana* and *S. carpocapsae* at the high dose. Seven days after exposure, no treatment exceeded 49% mortality. The maximum observed mortality was observed for the combination of *B. bassiana* and the high dose of *S. carpocapsae* (48.64%). After 14 days of exposure, the combined treatment of *B. bassiana* and *S. carpocapsae* at both high and low doses exhibited statistically higher mortalities than treatments alone. The maximum observed mortality was 70.74% for the application of *B. bassiana* and *S. carpocapsae* at the high dose, followed by the same combination at the low dose (56.84%), the single treatment of *S. carpocapsae* at 100 IJs cm^−2^ (35.26%) and 50 IJs cm^−2^ (25.78%), and *B. bassiana* alone (16.36%) (Table 7).

## 4. Discussion

Several factors, including environmental conditions, virulence of species or strain of EPF, type of grain, and susceptibility of target insect species/instar can impact the efficacy of EPF against insect pests [73,74,75,76,77]. In the present study, the EPF isolate of *B. bassiana* (WG-13) exhibited varying effects on the mortality of the storage pests included in the bioassays. *Beauveria bassiana* was the most effective against *R. dominica*, followed by *S. oryzae*, *T. castaneum*, *C. ferrugineus*, *O. surinamensis*, and *T. granarium*. Among all treatments, the single application of this EPF isolate was the least effective treatment. Wakil et al. [74], who studied four Pakistani strains of *B. bassiana* (WG-47, -48, -50, and -51), reported considerable variations in mortalities of *R. dominica* (26.2–42.4%), *Sitophilus granarius* (L.) (Coleoptera: Curculionidae) (24.5–38.4%), and *T. castaneum* (18.9–30.1%) at 14 days post-exposure, and *T. granarium* (23.0–35.9%) at 15 days post-exposure. The present study also found higher susceptibility of *R. dominica* to the EPF strain WG-13 used in this study, in line with the mortality range presented in the aforementioned research. This further reinforces the notion that different EPF strains exhibit diverse levels of efficacy against different stored-grain pest species, an issue that emphasizes the need for additional research aimed at uncovering the most virulent strains, to maximize their potential in commercial application [33].

EPNs have received moderate attention against post-harvest pests due to their requirement for elevated humidity conditions, rendering them unfit for the typically sere stored-product environment [43,78,79,80]. However, it is worth noting that a relative humidity of 65%, as used in this and former studies [67,81,82], is quite feasible in storage facilities, especially during the summer period [83]. Moreover, during summer, the populations of numerous stored-grain coleopterans occur at low numbers, while they subsequently increase from September to October [84,85,86]. Thus, by applying a combination of EPNs during the summer, this approach can potentially reduce the already diminished coleopteran populations, providing an added advantage in pest management strategies. Therefore, the discovery of highly virulent EPN strains has further enhanced their efficacy [64], garnering increased interest in their utilization against stored-grain pests. To date, the EPN, *S. carpocapsae*, constitutes one of the most effective EPNs used against insect pests [67,79,81,82,87], as it constitutes a vector for enteric entomopathogenic bacteria of the genus *Xenorhabdus* [88]. Some of its advantageous characteristics for the management of insect pests as a nematode species are its high fecundity, small size, and short generation time, as well as its safety concerning human health, the environment, and non-target organisms [67,88,89]. Based on their foraging strategy, EPNs are classified into two broad categories, ambushers (sit and wait) and cruisers (widely foraging) [90]. Cruisers have a higher likelihood of locating stationary and hidden resources compared to ambushers, while ambushers are more efficient at finding resources that have high mobility [90]. *Steinernema carpocapsae* is widely characterized as an ambush forager, however, this hypothesis has been contested due to a lack of evidence [91]. According to Wilson et al. [91], due to the adaptation to habitats other than mineral soils, such as peat, leaf litter, or wood, it is also capable of cruising long distances toward hosts, behaving as both an ambusher and a cruiser in such environments. The cruising behavior of *S. carpocapsae* has also been recently confirmed by Ebrahimi et al. [92], who studied the efficacy of *S. carpocapsae* on *Phthorimaea operculella* (Zeller) (Lepidoptera: Gelechiidae). Additionally, Baiocchi et al. [93] observed that the host-seeking behavior of *S. carpocapsae* was enhanced when stimulated with the physical contact of the host cuticle. Here, *S. carpocapsae* exhibited a ranging effect on the mortality of the storage pests included in the bioassays. *Steinernema carpocapsae* was the most effective against *R. dominica*, followed by *S. oryzae*, *T. castaneum*, *T. granarium*, *C. ferrugineus*, and *O. surinamensis*. *Cryptolestes ferrugineus* is considered a highly mobile species compared to *R. dominica* [94,95,96]. This leads to the hypothesis that *S. carpocapsae* might have adopted the cruising foraging behavior, at least in the substrate used in the current study, leading to higher mortality rates for the pest species characterized by lower mobility (*R. dominica*) compared to those with higher mobility (*C. ferrugineus*). Alikhan et al. [97] were pioneers in the observation of the impact of *S. carpocapsae* on major stored-product insects, such as *S. granarius*, *Tribolium confusum* Jacquelin du Val (Coleoptera: Tenebrionidae), and the larvae of *T. granarium*, but reported low levels of mortality. Ramos-Rodrıguez et al. [79] studied the efficacy of three EPNs of the genus *Steinernema*, including *S. carpocapsae*, against eight common stored-product coleopteran pests on filter paper. The results suggested that the percentage of mortality varied depending on the EPN species, the insect pest species, and the insect life stage. However, the authors found *R. dominica* and *S. oryzae* less susceptible than *T. castaneum* and *O. surinamensis*, contradicting the findings of this study. Our results are in line with Trdan et al. [44], who studied the efficacy of four EPNs against *Sitophilus granarius* (L) (Coleoptera: Curculionidae) and *O. surinamensis* on filter paper. They reported that the mortality of *O. surinamensis* was 15.79% and 21.05% when exposed to *S. carpocapsae* for seven days at 25 °C at two different concentrations at 500 and 1000 IJs mL^−1^, respectively. In a recent study, Erdoğuş et al. [98] revealed high susceptibility of *T. castaneum* to the same EPN species in arenas containing wheat crumbs. Therefore, there is no general tendency about the virulence of *S. carpocapsae* when used for the control of certain storage insects. Taking the current and aforementioned studies into account, insect developmental stage, dose, and used protocols are important parameters affecting the obtained results of each research.

EPNs have been evaluated in combination with insecticides, biocontrol agents, and parasitoids to increase their efficacy against insect pests [49,99,100,101,102,103]. Several factors can influence the interaction between EPF and EPNs, such as the species and strains of EPF and EPNs, the target insect host, application parameters, and environmental conditions [49,103,104,105,106,107]. The combined use of two biocontrol agents can result in diverse effects on the mortality of the target pests [49,53,89,108,109,110]. For example, additive effects occur when the biocontrol organisms act independently without interaction, while antagonism occurs when there is interaction between the two organisms. Strong competition for resources can occur when EPF and EPNs infest an insect in tandem. In antagonistic interactions, the growth of the EPF can be inhibited by secondary metabolites of *Photorhabdus* spp. and *Xenorhabdus* spp. bacteria carried by the EPNs, which exhibit an antifungal activity [111]. EPF naturally produce various toxic metabolites to eliminate their host insects [112]. Nevertheless, certain EPF compounds have also been found to possess antibiotic properties [54,113]. These adaptations of each biocontrol agent to the other result in a smaller-than-expected effect when they are combined compared to the total effect of both agents [49,89]. Nevertheless, the simultaneous infection of insects with both EPF and EPNs can enhance the efficacy of both organisms in biological control. The EPF infection induces stress in the host, resulting in a disruption of food intake and overall homeostasis, reduction of locomotion, and increased irritability [49,105,111,114,115]. These effects impair the host’s ability to resist nematode infection, which is typically effective in healthy insects [111,116]. Debilitated EPF-infected insects respire more, attracting EPNs that follow a path of CO_2_ toward their hosts [52,106]. *Steinernema feltiae* and *H. bacteriophora* have been reported to disperse conidia and blastospores of *Isaria fumosorosea* in agar dishes and soil, suggesting a high ability for EPNs to spread EPF conidia in the shared environment [117]. These mechanisms of interaction can lead EPF and EPNs to generate a stronger effect than the sum of their individual effects [49,89].

Various combinations of EPF and EPN species have previously been tested against several insect pests, exhibiting positive or negative effects in terms of pest mortality [52,53,54,56,107,109,110]. Here, the combination of *B. bassiana* and *S. carpocapsae* was not detrimental when applied to all tested insect species. Barbercheck and Kaya [50] found that this biocontrol agent combination resulted in additive effects against *Spodoptera exigua* (Hübner) (Lepidoptera: Noctuidae). Shapiro-Ilan et al. [107] observed an additive or antagonistic effect of *B. bassiana* and *S. carpocapsae* against *Curculio caryae* Horn (Coleoptera: Curculionidae) depending on the application doses, as observed again later by Ibrahim [118] for *Agrotis ipsilon* (Hufnagel) (Lepidoptera: Noctuidae). In more recent literature, additive effects of the *B. bassiana* and *S. carpocapsae* combination have been reported for *Hylobius abietis* (L) (Coleoptera: Curculionidae) [119,120], *Rhagoletis pomonella* (Walsh) (Diptera: Tephritidae) [121], *Rhynchophorus ferrugineus* (Olivier) (Coleoptera: Curculionidae) [122], *Bactrocera zonata* (Saunders) (Diptera: Tephritidae), and *Bactrocera dorsalis* (Hendel) (Diptera: Tephritidae) [62]. Regarding the joint use of EPF and EPNs against stored-grain pests, first investigations into the concurrent use of EPF and EPN species exhibited that the joint application of *B. bassiana* and *S. carpocapsae* on larvae of *T. castaneum* had an antagonistic interaction [57]. Furthermore, the joint use of *S. carpocapsae* and *C. farinosa* revealed an antagonistic effect on *T. granarium* and *T. castaneum* [58]. The additive effect found in this study when *B. bassiana* plus *S. carpocapsae* were applied against *T. castaneum* could be attributed to the fact that, in our study, we used adults of this species vs. larvae in Kamionek et al. [57]. This fact could be partially explained, regarding *S. carpocapsae*, since this EPN exhibits different virulence when applied on larvae and adults of *T. granarium* [67], even within larval instars [82]. Similarly, larvae and adults of *T. confusum* suffered different mortalities when exposed to various doses of *S. carpocapsae* [81]. Further experimental efforts are needed to justify this hypothesis.

## 5. Conclusions

The present study demonstrated that the concurrent application of *B. bassiana* and *S. carpocapsae* has yielded statistically higher mortality rates against all stored-product pests tested compared to the single treatment of each biocontrol agent. The combined use of this EPF and EPN shows promising potential as a viable treatment method against storage pests, lacking antagonistic interactions. Nevertheless, this particular combination has been poorly investigated in the realm of stored products. Therefore, to optimize its effectiveness, further research is imperative for the implementation of this biocontrol approach. Diverse strains and combinations of *B. bassiana* and *S. carpocapsae*, including other species/strains of EPF and EPN, along with varying biotic and abiotic conditions, should be examined against a range of stored-grain pests and their developmental stages. Additionally, careful consideration should be given to assessing the optimal mode of application and evaluating the economic feasibility of the treatment.

## Figures and Tables

**Table 1:** ANOVA parameters for the mortality of *R. dominica*, *S. oryzae*, *T. castaneum*, *C. ferrgineus*, *T. granarium*, and *O. surinamensis* when exposed to single and combined treatments of *B. bassiana* and *S. carpocapsae*. Total df = 89.

Effect	df	*R. dominica*		*S. oryzae*		*T. castaneum*		*T. granarium*		*C. ferrugineus*		*O. surinamensis*	
		*F*	*p*-Value	*F*	*p*-Value	*F*	*p*-Value	*F*	*p*-Value	*F*	*p*-Value	*F*	*p*-Value
Treatment	4	175.2	<0.01	202.3	<0.01	186.4	<0.01	110.7	<0.01	141.5	<0.01	117.1	<0.01
Interval	2	360.8	<0.01	447.1	<0.01	410.9	<0.01	365.8	<0.01	304.1	<0.01	231.8	<0.01
Treatment × Interval	8	12.7	<0.01	17.8	<0.01	16.4	<0.01	13.2	<0.01	15.0	<0.01	12.3	<0.01

**Table 2:** Mean mortality (% ± SE) of *R. dominica* at 3, 7, and, 14 days post-exposure when treated with two different concentrations of *S. carpocapsae* (Sc1: 50 IJs cm^−2^ and Sc2: 100 IJs cm^−2^) and *B. bassiana* (Bb: 1 × 10^6^ conidia mL^−1^) alone and in combination. Means followed by the same lower-case letters within each column are not significantly different (In all cases df = 4,29, Tukey–Kramer (HSD) test at *p* = 0.05). Means followed by the same upper-case letters within each row are not significantly different (In all cases df = 2,17, Tukey–Kramer (HSD) test at *p* = 0.05).

Treatment	Exposure				
	3 Days	7 Days	14 Days	*F*	*p*-Value
Sc1	15.13 ± 2.23 Ccd	32.23 ± 2.85 Bcd	43.59 ± 1.74 Acd	38.0	<0.01
Sc2	22.63 ± 3.03 Cbc	36.36 ± 2.53 Bc	51.22 ± 43 Ac	34.7	<0.01
Bb	8.42 ± 2.12 Cd	25.43 ± 1.32 Bd	36.71 ± 3.19 Ad	37.0	<0.01
Sc1 + Bb	26.97 ± 3.24 Cab	58.42 ± 2.18 Bb	82.85 ± 1.79 Ab	127.0	<0.01
Sc2 + Bb	34.47 ± 3.03 Ca	71.22 ± 2.36 Ba	96.62 ± 1.67 Aa	166.0	<0.01
*F*	13.3	69.6	160.0	-	-
*P*	<0.01	<0.01	<0.01	-	-

**Table 3:** Mean mortality (% ± SE) of *S. oryzae* at 3, 7, and 14 days post-exposure when treated with two different concentrations of *S. carpocapsae* (Sc1: 50 IJs cm^−2^ and Sc2: 100 IJs cm^−2^) and *B. bassiana* (Bb: 1 × 10^6^ conidia mL^−1^) alone and in combination. Means followed by the same lower-case letters within each column are not significantly different (In all cases df = 4,29, Tukey–Kramer (HSD) test at *p* = 0.05). Means followed by the same upper-case letters within each row are not significantly different (In all cases df = 2,17, Tukey–Kramer (HSD) test at *p* = 0.05).

Treatment	Exposure				
	3 Days	7 Days	14 Days	*F*	*p*-Value
Sc1	12.71 ± 2.88 Cbc	29.86 ± 1.86 Bcd	38.86 ± 1.96 Ad	33.8	<0.01
Sc2	18.55 ± 2.70 Cab	32.45 ± 2.05 Bc	46.57 ± 0.49 Ac	49.9	<0.01
Bb	6.75 ± 1.70 Cc	21.31 ± 1.95 Ad	32.71 ± 2.05 Ad	46.4	<0.01
Sc1 + Bb	23.72 ± 1.65 Ca	52.01 ± 2.89 Ab	75.83 ± 1.79 Ab	142.0	<0.01
Sc2 + Bb	27.85 ± 2.62 Ca	65.00 ± 1.31 Ba	90.48 ± 1.63 Aa	263.0	<0.01
*F*	12.6	73.8	220.0	-	-
*P*	<0.01	<0.01	<0.01	-	-

**Table 4:** Mean mortality (% ± SE) of *T. castaneum* at 3, 7, and 14 days post-exposure when treated with two different concentrations of *S. carpocapsae* (Sc1: 50 IJs cm^−2^ and Sc2: 100 IJs cm^−2^) and *B. bassiana* (Bb: 1 × 10^6^ conidia mL^−1^), alone and in combination. Means followed by the same lower-case letters within each column are not significantly different (In all cases df = 4,29, Tukey–Kramer (HSD) test at *p* = 0.05). Means followed by the same upper-case letters within each row are not significantly different (In all cases df = 2,17, Tukey–Kramer (HSD) test at *p* = 0.05).

Treatment	Exposure				
	3 Days	7 Days	14 Days	*F*	*p*-Value
Sc1	10.08 ± 1.29 Cbc	25.35 ± 1.63 Bd	36.62 ± 2.37 Ac	53.2	<0.01
Sc2	14.25 ± 2.98 Bbc	34.64 ± 2.49 Bbc	43.55 ± 2.32 Ac	33.0	<0.01
Bb	5.00 ± 1.29 Cc	19.47 ± 1.97 Ad	27.28 ± 1.98 Ad	40.3	<0.01
Sc1 + Bb	19.25 ± 2.65 Cab	48.28 ± 2.05 Bab	71.71 ± 2.00 Ab	136.0	<0.01
Sc2 + Bb	26.79 ± 2.67 Ca	60.17 ± 1.64 Ba	87.23 ± 1.63 Aa	229.0	<0.01
*F*	13.3	72.5	146.0	-	-
*P*	<0.01	<0.01	<0.01	-	-

**Table 5:** Mean mortality (% ± SE) of *T. granarium* at 3, 7, and 14 days post-exposure when treated with two different concentrations of *S. carpocapsae* (Sc1: 50 IJs cm^−2^ and Sc2: 100 IJs cm^−2^) and *B. bassiana* (Bb: 1 × 10^6^ conidia mL^−1^) alone and in combination. Means followed by the same lower-case letters within each column are not significantly different (In all cases df = 4,29, Tukey–Kramer (HSD) test at *p* = 0.05). Means followed by the same upper-case letters within each row are not significantly different (In all cases df = 2,17, Tukey–Kramer (HSD) test at *p* = 0.05).

Treatment	Exposure				
	3 Days	7 Days	14 Days	*F*	*p*-Value
Sc1	1.66 ± 1.66 Cbc	9.34 ± 1.50 Bc	29.25 ± 2.00 Ac	50.2	<0.01
Sc2	4.21 ± 1.54 Cbc	21.31 ± 1.91 Bb	33.55 ± 1.95 Abc	48.1	<0.01
Bb	0.00 ± 0.00 Cc	5.87 ± 2.38 Bc	13.77 ± 1.03 Ad	21.2	<0.01
Sc1 + Bb	7.54 ± 2.13 Cab	22.19 ± 1.63 Bb	41.36 ± 1.75 Bb	83.3	<0.01
Sc2 + Bb	12.63 ± 2.19 Ca	35.08 ± 1.68 Ba	57.71 ± 1.69 Ba	151.0	<0.01
*F*	9.0	39.5	60.3	-	-
*P*	<0.01	<0.01	<0.01	-	-

**Table 6:** Mean mortality (% ± SE) of *C. ferrugineus* at 3, 7, and 14 days post-exposure when treated with two different concentrations of *S. carpocapsae* (Sc1: 50 IJs cm^−2^ and Sc2: 100 IJs cm^−2^) and *B. bassiana* (Bb: 1 × 10^6^ conidia mL^−1^) alone and in combination. Means followed by the same lower-case letters within each column are not significantly different (In all cases df = 4,29, Tukey–Kramer (HSD) test at *p* = 0.05). Means followed by the same upper-case letters within each row are not significantly different (In all cases df = 2,17, Tukey–Kramer (HSD) test at *p* = 0.05).

Treatment	Exposure				
	3 Days	7 Days	14 Days	*F*	*p*-Value
Sc1	5.00 ± 2.58 bc	18.72 ± 2.28 Acd	27.23 ± 2.60 Ad	20.3	<0.01
Sc2	9.25 ± 2.71 Cbc	26.22 ± 1.89 Abc	38.42 ± 2.00 Ac	42.9	<0.01
Bb	2.54 ± 1.13 Bc	11.88 ± 2.87 ABd	19.56 ± 2.92 Ad	10.2	<0.01
Sc1 + Bb	13.46 ± 2.13 Cab	39.78 ± 1.77 Bb	61.57 ± 2.00 Ab	149.0	<0.01
Sc2 + Bb	18.50 ± 1.12 Ca	51.71 ± 2.05 Ba	76.05 ± 1.70 Aa	298.0	<0.01
*F*	9.8	52.9	107.0	-	-
*P*	<0.01	<0.01	<0.01	-	-

**Table 7:** Mean mortality (% ± SE) of *O. surinamensis* at 3, 7, and 14 days post-exposure when treated with two different concentrations of *S. carpocapsae* (Sc1: 50 IJs cm^−2^ and Sc2: 100 IJs cm^−2^) and *B. bassiana* (Bb: 1 × 10^6^ conidia mL^−1^) alone and in combination. Means followed by the same lower-case letters within each column are not significantly different (In all cases df = 4,29, Tukey–Kramer (HSD) test at *p* = 0.05). Means followed by the same upper-case letters within each row are not significantly different (In all cases df = 2,17, Tukey–Kramer (HSD) test at *p* = 0.05).

Treatment	Exposure				
	3 Days	7 Days	14 Days	*F*	*p*-Value
Sc1	4.16 ± 2.71 Cb	16.22 ± 4.37 Bcd	25.78 ± 2.13 Acd	24.6	<0.01
Sc2	8.55 ± 1.79 Cab	23.02 ± 3.04 Bc	35.26 ± 2.57 Ac	28.0	<0.01
Bb	2.54 ± 2.23 Bb	9.38 ± 1.53 ABd	16.36 ± 2.05 Ad	12.4	<0.01
Sc1 + Bb	11.75 ± 3.06 Cab	35.92 ± 3.03 Ab	56.84 ± 1.89 Ab	68.8	<0.01
Sc2 + Bb	17.76 ± 2.48 Ca	48.64 ± 1.48 Ba	70.74 ± 2.77 Aa	132.0	<0.01
*F*	6.0	48.6	94.0	-	-
*P*	<0.01	<0.01	<0.01	-	-

## Data Availability

Data are available within the article.
